# One-step Preparation of a VHH-based Immunoadsorbent for the Extracorporeal Removal of β2-microglobulin

**DOI:** 10.3390/molecules24112119

**Published:** 2019-06-04

**Authors:** Lijun Zhang, Berlin Zang, Chundong Huang, Jun Ren, Lingyun Jia

**Affiliations:** Liaoning Key Laboratory of Molecular Recognition and Imaging, School of Bioengineering, Dalian University of Technology, No.2 Linggong Road, Dalian, Liaoning 116023, China; zhanglijun1022@mail.dlut.edu.cn (L.Z.); dutberlin@mail.dlut.edu.cn (B.Z.); cd_huang_bio@mail.dlut.edu.cn (C.H.); renjun@dlut.edu.cn (J.R.)

**Keywords:** β2-microglobulin, VHH, formylglycine-generating enzyme, hemodialysis

## Abstract

Dialysis-related amyloidosis (DRA), which has been widely recognized to be associated with the accumulation of β2-microglobulin (β2-m) in blood, is one of the most common complications in patients receiving long-term dialysis treatment. The most significant side-effect of existing hemodialysis sorbents for the removal of β2-m from blood is the loss of vital proteins due to non-specific adsorptions. Although the traditional antibodies have the capability to specifically remove β2-m from blood, high cost limits their applications in clinics. Single domain antibodies derived from the Camelidae species serve as a superior choice in the preparation of immunoadsorbents due to their small size, high stability, amenability, simplicity of expression in microbes, and high affinity to recognize and interact with β2-m. In this study, we modified the anti-β2-m VHH by the formylglycine-generating enzyme (FGE), and then directly immobilized the aldehyde-modified VHH to the amino-activated beads. Notably, the fabrication is cost- and time-effective, since all the preparation steps were performed in the crude cell extract without rigorous purification. The accordingly prepared immunoadsorbent with VHHs as ligands exhibited the high capacity of β2-m (0.75 mg/mL). In conclusion, the VHH antibodies were successfully used as affinity ligands in the preparation of novel immunoadsorbents by the site-specific immobilization, and effectively adsorbed β2-m from blood, therefore opening a new avenue for efficient hemodialysis.

## 1. Introduction

The kidney plays a central role in the removal of metabolic wastes from our bloodstream by making urine. However, chronic kidney disease, which involves persistent kidney injury or glomerular filtration rate (GFR) lower than 60 mL/min per 1.73 m^2^ for 90 days or longer, has been proven to be a primary fast-growing health problem around the world, contributing to the rising of kidney failure, cardiovascular disease (CVD), and premature death [1,2,3].

β2-Microglobulin (β2-m), a highly conserved globular protein (11.8 kDa), is a major middle-molecular uremic toxin. β2-m is a component of the major histocompatibility complex on almost all nucleated cell membranes, which can detach from the membrane and eventually be catabolized in renal tubules. However, the pathological concentration of β2-m in hemodialysis patients might rise to a level that is 50 times higher than that under normal conditions [4]. Traditional hemodialysis devices fail to eliminate β2-m from blood, and thus lead to the long-term accumulation of β2-m in serum or systemic organs [5]. The resulting deposits have been identified as dialysis-related amyloidosis (DRA) [6]. Symptoms in patients with DRA [7,8] mainly consist of joint pain, carpal tunnel syndrome (CTS), destructive spondyloarthropathy (DSA), and cystic bone lesions with associated fractures, which tremendously reduce patients’ activity of daily living (ADL) [9]. Therefore, it is of vital importance to reduce the serum β2-m concentration to healthy levels clinically. The aggressive removal of β2-m has been proven useful in the prevention and remission of DRA [10]. Although several hemodialysis devices mimic the functions of kidney by hemodialysis, hemodiafiltration, and peritoneal dialysis, most of these non-specific technologies are unable to reach clinical remission [11].

Developing a high-specific adsorbent for β2-m remains a central issue according to clinical assessments of different patients [12,13]. The first listed β2-m selective adsorbent, Lixelle ^TM^ (Kaneka Co., Ltd., Osaka, Japan) [14], was designed to adsorb β2-m through the combination of hydrophobic interaction and applicable pore size. With the hydrophobic hexadecyl groups immobilized on the cellulose beads, Lixelle adsorbed not only β2-m but also insulin, lysozyme, and retinol-binding proteins [15]. An in vitro study showed that inflammatory cytokines, such as interleukins IL-6 and IL1-β, were also adsorbed to the column [16]. To acquire a high β2-m binding specificity, antibodies serve as superior candidates for the innovative hemodialysis strategies. The monoclonal antibody and scFv-based immunoadsorbents [17] were fabricated recently for the removal of β2-m without causing significant loss of valuable proteins conducive to patients [18]. By possessing both high affinity to β2-m and fast-apparent rate of adsorption, which are essential in consideration of the limited treatment time, the antibody-based immunoadsorbents could efficiently reduce the concentration of β2-m to a healthy level in blood [18]. Notwithstanding, the low stability and the high cost of the synthetic affinity ligands limits the applications of monoclonal antibody or scFv-based immunoadsorbents. Therefore, the development of cost-efficient affinity ligands with high-selectivity is of urgent need.

The VHHs, also referred to as single-domain antibodies or nanobodies [19], possess a small size (about 15 kDa, 1/10 of human IgG) [20], leading to higher densities and available antigen-binding sites on the adsorbent compared to traditional antibodies. VHHs usually possess high stability, high affinity, and low immunogenicity [21]. Furthermore, the VHHs can be obtained from *Escherichia coli* or yeast with high yields, which would potentially facilitate the cost-effective large-scale production of VHHs. These advantages of VHH antibodies, combined with the explicit affinity to β2-m, encouraged the attempted fabrication of a novel VHH-based immunoadsorbent.

Here we present a facile one-step method for the preparation of a VHH-based immunoadsorbent using the formylglycine-generating enzyme to introduce a site-specific aldehyde group that facilitates the subsequent site-specific immobilization at the C-terminus of VHH. The novel adsorbent showed a high specificity to β2-m. Meanwhile, combinatorial approaches based on VHH antibodies open a new avenue to the rapid, feasible, and low-cost synthesis of new synthetic affinity ligands of precise utilization with scale-up potential in the novel immunoadsorbent preparation.

## 2. Results and Discussion

### 2.1. Expression of Recombinant VHHs and FGE

**Figure 1:**
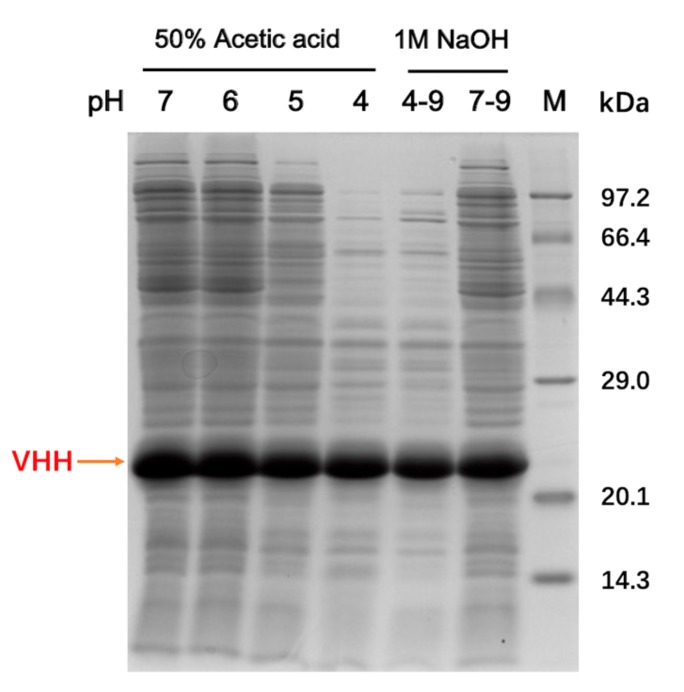
Acetic acid-assisted precipitation of host cell proteins. Lane M is the protein molecular weight marker; 50% acetic acid solution was added dropwise into the crude cell extract. The supernatant treated with different pH values were analyzed by SDS-polyacrylamide gel electrophoresis (SDS-PAGE) and gray scanning. After precipitation, the supernatant was readjusted to pH 9 (Lanes 4–9). The lysate, which directly adjusted from pH 7 to pH 9, was set as a control (Lanes 7–9).

A previous study confirmed a low productivity when aldehyde-tagged protein A was co-expressed with FGE to achieve the conversion of cysteine to formylglycine in vivo [22]. In this work, aldehyde-tagged VHH and histidine-tagged FGE were expressed in *E. coli* shuffle T7 and *E. coli* BL21 (DE3) separately to improve the expression level of each protein. The yield of the VHH was about 400 mg per liter of cell culture. The purity of soluble VHHs in the cell extract was largely improved to about 60% after the acid-assisted precipitation of endogenous proteins, which was relatively conducive to the subsequent catalysis process (Figure 1).

FGE was expressed in *E. coli* BL21 (DE3) in soluble form, and the yield of FGE was about 300 mg per liter of cell culture. Previous studies showed that Cu(II) played a crucial role in the catalytic activity of FGE [23,24,25,26], so 10 μM CuSO_4_ was added to the culture medium during the protein expression to improve the activity of FGE.

### 2.2. Validation of the Site-Specific Aldehyde Modification

**Figure 2:**
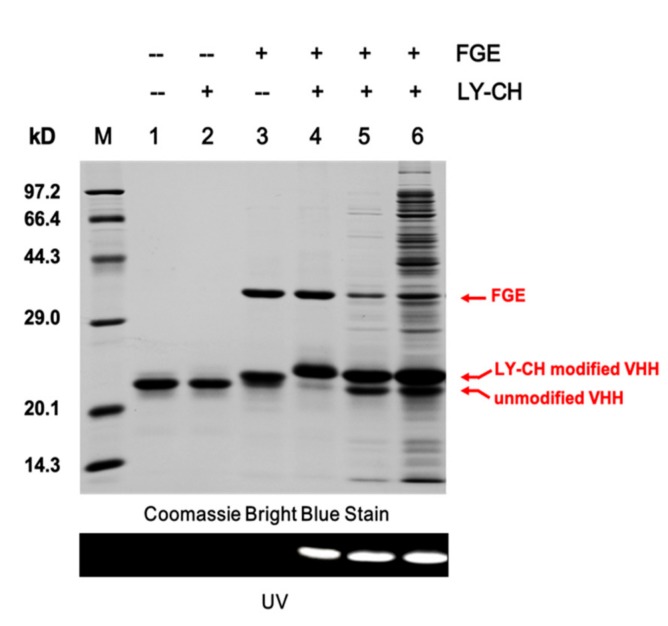
Validation of the site-specific modification of VHHs. SDS-PAGE analysis of Aldtag-VHH and the lucifer yellow CH (LY-CH) labeled products. Lane M, molecular weight markers; lane 1, native Aldtag-VHH; lane 2, Aldtag-VHH incubated with LY-CH; lane 3, FGE modified Aldtag-VHH incubated without LY-CH; lane 4, FGE modified Aldtag-VHH incubated with LY-CH; lane 5, crude cell extract was pretreated with 50% acetic acid to pH 4 with adjustment to pH 9 with 1 M NaOH; lane 6, crude cell extract pretreated with 1 M NaOH (adjusted directly to pH 9).

**Figure 3:**
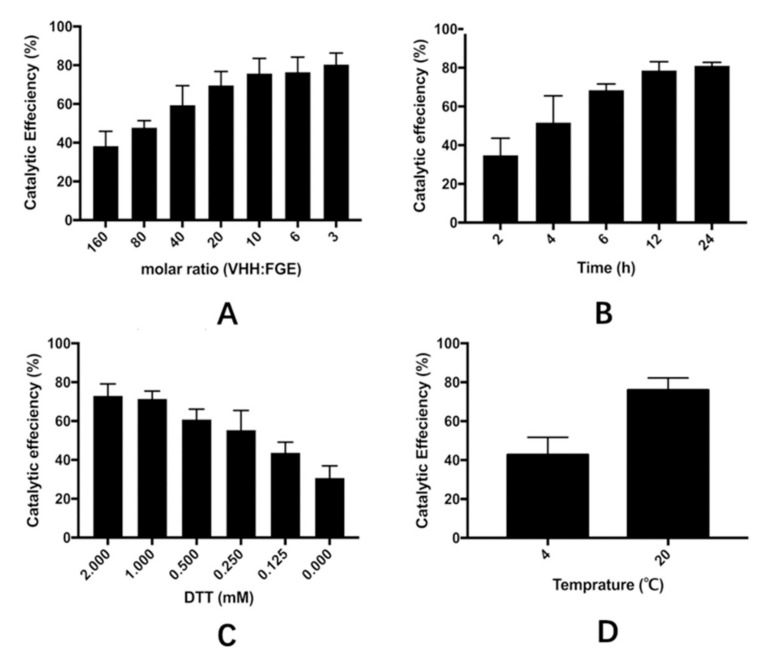
Optimization of FGE-catalytic conditions of VHH catalysis in the crude cell extract. Four factors were investigated, including (**A**) the molar ratio of VHH and FGE, (**B**) incubation time, (**C**) the concentration of DTT added to the catalytic buffer, and (**D**) the catalytic temperature. The products of VHH incubated with FGE under different conditions were analyzed by SDS-PAGE, and the catalytic efficiency was calculated using the software of ImageJ. Data are presented as means ± SDs, *n* = 3. Error bars are within symbol size if not visible.

**Figure 4:**
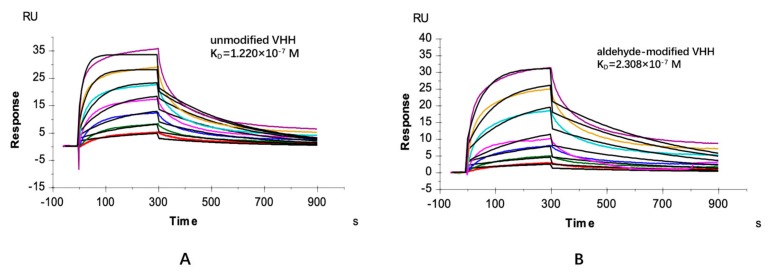
Affinities of the unmodified (**A**) and modified VHHs (**B**) binding β2-m investigated by Biacore T200. For surface plasmon resonance spectroscopy (SPR)-based affinity measurements, β2-m was covalently coupled on a CM5-chip. Kinetic measurements were performed by injecting seven concentrations ((162.8, 81.40, 40.70, 20.35, 10.17, 5.087, 2.544) × 10^−8^ M) of purified VHHs. The obtained data sets were evaluated using the 1:1 Langmuir binding model.

A hydrazide-containing dye, Lucifer Yellow CH (LY-CH), was applied as a probe to validate the generation of the aldehyde group on the VHH fragment from the crude cell extract after catalysis, as hydrazide selectively reacts with aldehyde under acidic condition via hydrazone ligation. The labeled product was analyzed by SDS-PAGE, and the amount of aldehyde modified VHHs was calculated by gray scanning. As shown in Figure 2, VHH antibodies modified by FGE exhibited strong fluorescence under UV-light when labeled with LY-CH (Figure 2, Lanes 4–6). The yield of modified VHHs from the cell extract (61.4%, Lane 4) was lower than that from the purified VHH/FGE mixture (90.4%, Lane 5) under the same catalytic condition, possibly attributable to the disturbance of host cell proteins in cell extract, which hinder the interaction between FGE and the aldehyde tag on the VHH. An increase in the conversion efficiency was obtained by extending incubation time or increasing the dosage of FGE (Figure 3). Finally, the conditions were optimized to be: molar ratio of VHH and FGE of 10:1, concentration of DTT of 2 mM, catalytic temperature of 20 °C, incubation time of 12 h, with catalytic efficiency of up to nearly 80%, which are able to accomplish the subsequent coupling process. It was also demonstrated that the 6-mer amino acids sequence (LCTPSR) attached to the C-terminus of the anti-β2-m VHH was well recognized by FGE. Besides, there was no significant difference between the affinity constants of the modified and the unmodified VHH as measured by Biacore T200 (Figure 4), indicating that the aldehyde modification on VHHs did not harm the conformation or the activity of the protein of interest.

### 2.3. β2-m Adsorption Performance of the VHH Immunoadsorbent

During the immobilization process, about 54% of the total VHHs in the cell extract had been coupled onto agarose beads using the amino-activated Sepharose CL-6B, which was previously synthesized at a ligand density of 12 μmol/mL. The gel was washed with adequate PBS and 1 M NaCl solution. The protein bound on the resin was calculated by comparing the VHH concentration before and after immobilization and the VHH density was determined as 1.2 ± 0.3 mg/mL gel.

**Figure 5:**
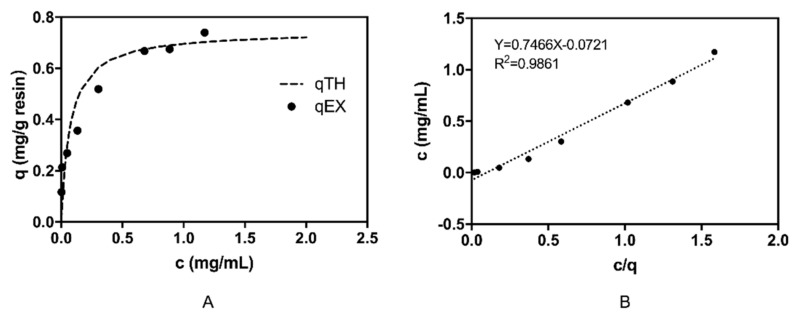
The static adsorption performance of the VHH-based adsorbent in plasma. (**A**) The isothermal adsorption curve of the sorbent at room temperature; qEX represents the test data while qTH represents the theoretical data. (**B**) The rearranged linearization curve. The slope of the curve was the maximal adsorptive capacity of the gel, and the intercept of curve was the opposite number of *Kd*. Y represents the equilibrium concentration of β2-m; X represents *c/q* (see Equation 2 in Method 3.6).

As shown in Figure 5, the adsorption of the sorbent to β2-m was well-fitted with the Langmuir adsorption isotherm. The correlation coefficient (R^2^) of the rearranged Langmuir adsorption isotherm model for β2-m was 0.986, indicating that the adsorption of β2-m on the VHH-based immunoadsorbent was consistent with the Langmuir adsorption isotherm model, and the adsorption process was a monolayer process. Meanwhile, the VHH-based immunoadsorbent exhibited several promising characteristics. The maximal adsorptive capacity of the gel was 0.7466 mg β2-m/ mL settled gel and the *K_D_* value was 6.11 × 10^−6^ M according to the fitting equation.

Reports showed that 1 mg of the conventional IgG antibody could adsorb 0.16 mg of β2-m, while 1 mg of scFv fragment could bind 0.4 mg of β2-m [17]. When it comes to the VHHs, the theoretical binding capacity of β2-m for 1 mg of VHH (Mr = 1.83 kDa) could reach to 0.63 mg. The β2-m binding capacity of the VHH-based adsorbent was better than that of the traditional antibody-based mediums (consisting of traditional whole antibodies or scFv fragments), as investigated in previous studies [17,27]. Although the monoclonal antibodies or scFv-based immunoadsorbent could exhibit a high affinity to β2-m, which was superior to the traditional hydrophobic chemical ligands [14,28], they usually present low β2-m adsorption site density resulting from the inactivation of antibodies by immobilization chemistry (such as cyanogen bromide activation of agarose), leading to limited a portion of immobilized antibodies capable of binding to β2-m. A previous report showed that the immunoadsorbent based on monoclonal antibody GB_5_C_7_ possessed a maximum binding capacity of 100-105 μg β2-m/mg of antibody [29]. Another monoclonal antibody-based immunoadsorptive media had a binding site density of only 30 μg β2-m per mL of settled gel [30]. This VHH-based immunoadsorbent, by contrast, had a significant increase in the β2-m adsorption capacity.

### 2.4. Specific Adsorption Performance of the VHH Immunoadsorbent

**Figure 6:**
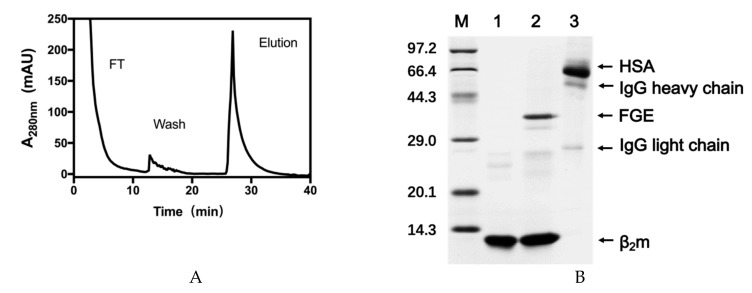
Adsorption of β2-m from β2-m enriched human serum using VHH-based immunosorbent fabricated through site-specific reductive amination conjugation. (**A**) Chromatography profile. (**B**) SDS-PAGE analysis of the chromatographic fractions. Lane 1, native β2-m; Lane 2, fraction eluted with 1% SDS; Lane 3, human serum. HSA represents the human serum albumin.

Dynamic binding capacity of β2-m from plasma was also investigated to evaluate the performance of the VHH-based sorbent. PBS containing 1 M NaCl was used in washing steps to remove nonspecific adsorption. The final sample eluted by 1% SDS from the gel was analyzed by SDS-PAGE and showed a good purity (Figure 6B, lane 2). However, a small quantity of FGE also appeared on the gel. FGE was not so stable in alkaline environment that the protein could precipitate on the gel during the coupling process. A purification step would be taken into account after catalysis to remove FGE in future production.

**Figure 7:**
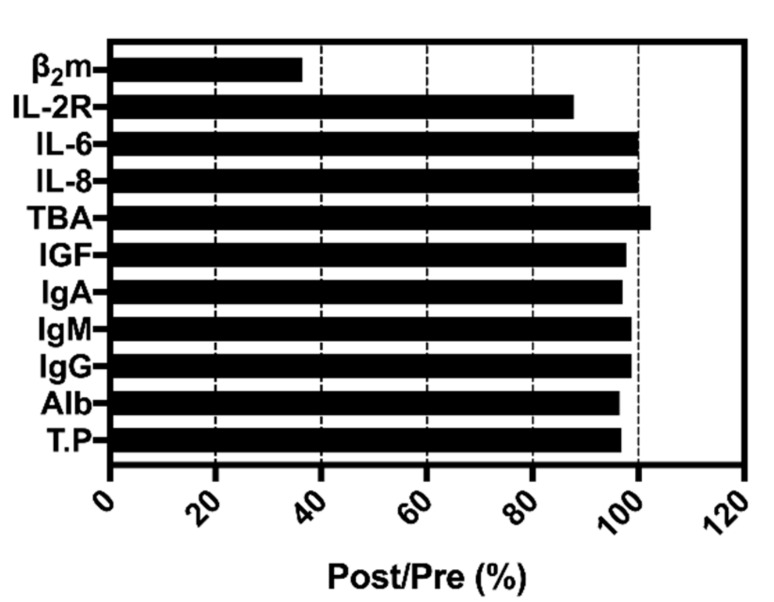
Changes in concentration of serum components before and after the adsorption balance in vitro. T.P, total protein; Alb, albumin; IGF, insulin-like growth factor; TBA, total bile acid; IL-6, interleukin 6; IL-8, interleukin 8; IL-2R, interleukin 2 receptor.

Another in vitro study was performed using the serum to evaluate the non-specific adsorption of the VHH-based immunoadsorbent. It was demonstrated that the matrix exhibited low adsorption to major proteins in blood (shown in Figure 7), which may due to the fact that the binding of β2-m to VHH on the beads was based on the specific interaction between antibody and antigen. Although these results proved the specific binding of the adsorbent to β2-m, future studies will focus more on screening VHHs with higher affinity to β2-m from the phage VHH library for the more efficient removal of β2-m from blood.

### 2.5. Storage Stability of the VHH-based Immunoadsorbent

**Figure 8:**
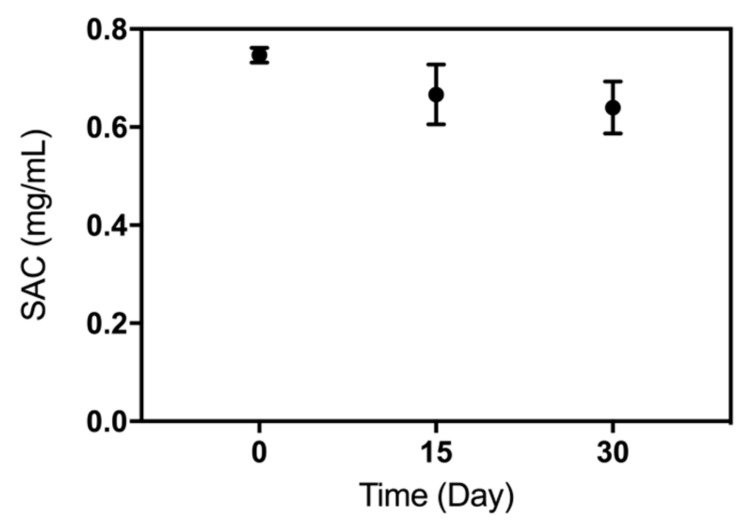
The saturation adsorption capacity (SAC) of the VHH-based immunoadsorbent preserved at 4 °C for one month (each experiment was performed three times).

The storage stability of the VHH adsorbent was also evaluated. The concentration of VHH that leaked from the gel was almost undetectable, suggesting the reductive amination reaction was a reliable immobilization method to be used on VHHs. Figure 8 shows that the β2-m binding capacity of the VHH-based immunoadsorbent still remained more than 80% if that of the freshly prepared sorbent was considered as 100% (*n* = 3). These results indicated that this one-step prepared VHH-based immunoadsorbent from the crude cell extract via site-specific immobilization was stable.

In summary, due to the facile production of the aldehyde-tagged proteins and readily accessible bioconjugate strategy, we fused aldehyde tags on the sequence of a VHH antibody, which had a high affinity towards β2-m. Then, the aldehyde loaded protein was introduced onto a DADPA-prepared sephorose to generate novel affinity adsorption resins, possessing a binding capacity of 0.75 mg β2-m per mL of settled gel. Furthermore, VHH antibodies can be easily expressed in microbial vectors and site-specifically modified without the loss of activity due to their stability and robustness, potentially reducing the production cost.

## 3. Materials and Methods

### 3.1. Reagents and Equipment

The plasmids pET21a and pET28a were obtained from Novagen (Madison, WI, USA). Strains *Escherichia coli* Shuffle T7, BL21 (DE3) were purchased from Takara Biotechnology (Dalian, China). Human β2-m was purchased from Crown Medical-Tech (Dalian) Co., Ltd. Sepharose CL-6B was purchased from GE Healthcare (Uppsala, Sweden). Lucifer Yellow CH probes (LY-CH) was purchased from Molecular Probes (Eugene, OR, USA). Human serum was obtained from Blood Bank of Dalian (Dalian, China). The 1,4-Butanediol diglycidyl ether was purchased from J&K Chemical (Beijing, China). The 2,2′,2-Nitrilotris (ethanol) was purchased from Aladdin Industrial Corporation. The 3,3′-diaminodipropylamine (DADPA) was purchased from Tokyo Chemical Industry (Tokyo, Japan). Double distilled water was obtained from a Milli-Q ultrapure water purification system (Millipore; Billerica, MA, USA).

### 3.2. Expression of VHH Antibody and FGE

The gene of the aldehyde-tagged VHH was cloned into the expression vector pET21a (Novagen) via the restriction sites *NdeI* and *XhoI*. The amino acid sequence encoding the 6×Histag and the aldehyde-tag (LCTPSR) was added to C-terminus of the VHH, registered as the plasmid pET21a-Aldtag-VHH. The plasmid was transferred into *E. coli* shuffle T7, which was then spread on the lysogeny broth (LB) agar plate (NaCl 10 g/L, tryptone 10 g/L, yeast extract 5 g/L, agar powder 1.5%) containing 100 μg/mL ampicillin. The plate was incubated at 37 °C overnight. A single positive clone was selected and cultured in LB for several hours until the OD_600_ reached 2.0. After that, terrific broth (TB) containing 100 μg/mL ampicillin was used for further culture in large scale. After a further incubation of cells in TB at 37 °C for 5 h, the expression of the aldehyde-tagged VHHs was introduced by the addition of 0.5 mM isopropyl β-D-thiogalactoside (IPTG). After cultivation for an additional 20 h at 18 °C, the cells were gathered by centrifugation at 8000 × *g* at room temperature for 20 min and then resuspended in 20 mM PBS (pH 7.4) containing 150 mM NaCl. The fragmentation of cells by a high-pressure homogenizer was performed for the extraction of Aldtag-VHH and the lysate was centrifuged at 10,000× *g* for 30 min. The clear supernatant was retained and designated as the crude cell extract.

The amino acid sequence encoding the 6×Histag was also added to C-terminus of the FGE. The sequence of the recombinant FGE was then constructed into the expression vector pET28a and transferred into an *E. coli* BL21 (DE3) strain. The expression of FGE depended on the similar culture conditions to VHHs, except for the use of kanamycin to replace ampicillin and the addition of 10 μM CuSO_4_ in terrific broth. Subsequently, FGE was purified by means of metal chelating chromatography.

### 3.3. Validation of Aldehyde on VHH

The cell extract was pretreated with 50% acetic acid solution to get rid of protein impurities on ice. After centrifugation, the buffer was adjusted to pH 9 with 1 M NaOH solution. Subsequently, the buffer solution was changed to reaction buffer (containing 50 mM 2,2′,2-Nitrilotris, 50 mM NaCl, and 1 mM DTT, pH 9) by buffer exchange using ultrafiltration devices. The catalysis procedure for FGE to contact the recognition site (LCTPSR) on VHH needed to be accomplished in alkaline condition. FGE was expressed and purified by means of metal chelating chromatography. FGE (10 mg/mL) and the VHH (about 3 mg/mL in cell extract, which was analyzed by SDS-PAGE and gray scanning) were mixed at a molar ratio of 1:10, and the mixture was incubated at room temperature for 6 h. Subsequently, a hydrazide-containing dye, LY-CH (10 mM), was applied to verify the successful generation of an aldehyde group on the VHH antibody. The 2 μL product after catalysis and 1 μL LY-CH were added to 18 μL 0.1 M acetate buffer (pH 4.0). The labeled mixture was incubated at 37 °C for 2 h. Then, 20 μL of the product was analyzed by SDS-PAGE using a 15% gel and the fluorescent image was obtained with an image recording system (Syngene, Cambridge, UK).

The affinity measurement of VHHs with or without modification towards β2-m was performed using surface plasmon resonance spectroscopy with a Biacore T200 instrument (GE Healthcare). Both VHHs were purified by means of metal chelating chromatography and gel filtration chromatography to meet the requirements of the purity of VHHs to be analyzed by SPR. β2-m was covalently immobilized on a CM5-chip (GE Healthcare) according to the standard protocol. β2-m was coupled to a response of 500 RU. Flow path 1 was activated and blocked to determine background. For kinetic measurement, seven concentrations ((162.8, 81.40, 40.70, 20.35, 10.17, 5.087, 2.544) × 10^−8^ M) of the VHHs were injected. The running buffer containing 10 mM PBS, 150 mM NaCl, and 0.05% surfactant P20 (pH 7.4) was used. For the association of VHHs, a flow rate of 30 μL/min for 300 s was applied, and for the dissociation a flow rate of 30 μL/min for 600 s was applied. A regeneration solution containing 100 mM Glycine-HCl (pH 2.0) was used. Measurements were performed at 25 °C. The data was evaluated using the biacore evaluation software.

### 3.4. Optimization of the Catalytic Process of VHH in the Cell Extract

In this study, four factors that influenced the yield of the aldehyde-modified VHH, including raw ratio, concentration of DTT, temperature, and incubation time, were investigated. VHH in the reaction buffer (containing 50 mM 2,2′,2-Nitrilotris, 50 mM NaCl, pH 9) was incubated with FGE at different molar ratios (160:1, 80:1, 40:1, 20:1, 10:1, 6:1, 3:1). The mixture was then incubated at 20 °C for 12 h after the addition of 2 mM DTT. Different concentrations (2, 1, 0.5, 0.25, 0.125, 0 mM) of DTT was added in the mixture of VHH and FGE (molar ratio of 10:1) before incubation at 20 °C for 12 h to determine the impact of the reductive reagent on the catalytic efficiency. Incubation time (from 2 h to 24 h) was also investigated in a mixture with a set molar ratio of VHH:FGE (10:1) and 2 mM DTT at 20 °C. In addition, temperatures during the incubation were set at 4 °C and 20 °C, respectively. All the products were analyzed by SDS-PAGE and the amounts of catalytic products were analyzed quantitatively using the software of ImageJ.

### 3.5. Fabrication of VHH-Based Immunoadsorbent

**Figure 9:**
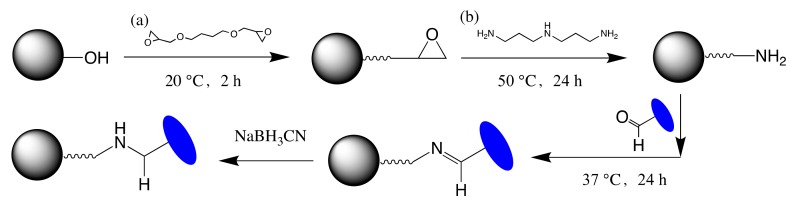
Scheme of the preparation of a VHH based immunoadsorbent: (**a**) 1,4-butanediol diglycidyl and (**b**) 3,3′-diaminodipropylamine were used to prepare the amino-activated matrix based on the CL-6B agarose beads. Aldehyde-modified VHHs were then coupled to the matrix via the reductive amination.

As shown in Figure 9, sepharose CL-6B (10 mL, purchased from GE Healthcare) was washed with water to remove storage preservatives and activated with 32%(*v/v*) 1,4-Butanediol diglycidyl ether in a mixture containing 0.35 M NaOH. The mixture was incubated at 20 °C for 2 h. The gel was washed with acetone, followed by further washing with plenty of water. The activated gel was then incubated with 10% 3,3′-diaminodipropylamine (DADPA) at 50 °C for 12 h to generate the amino group. After that, 20 mL 6% ethanolamine (pH 8.6) was added to the gel to block the remaining epoxy group.

Two milligrams of the catalysate were added into one milligram of the DADPA-modified gel, which was washed with the reaction buffer (pH 9). The coupling process was performed at 37 °C overnight, followed by washing with several bed volumes of coupling buffer. A further wash step containing 1 M NaCl was intended to remove nonspecific adsorption to the gel. Collection of all the washes and the amount of protein coupled could be calculated by the difference in the amount of protein present in the reaction medium before and after coupling. The Schiff base bond was reduced with sodium borohydride to prevent a leakage of VHH antibodies from the gel. Finally, the VHH-based immunoadsorbent was stored at 4 °C in 10 mM PBS containing 0.01% sodium azide.

### 3.6. Adsorption Isotherm in β2-m Enriched Human Serum

To investigate the adsorption isotherm of β2-m, the human blood serum containing different initial concentrations of β2-m (1.3, 1.0, 0.8, 0.4, 0.2, 0.1, 0.05, 0.025 mg/L) was used. The gel was washed with PBS and 100 μL gel, and was incubated with 500 μL of β2-m-enriched serum at room temperature under consistent rolling for 2 h. An immunoturbidimetry method was used to determine the concentration of β2-m before and after adsorption. The β2-m-adsorption capacity and *K_d_* of the novel VHH immunoadsorbent were subsequently calculated, using Langmuir adsorption isotherm (Equation 1) and its rearranged equation (Equation (2)).

(1) $$q=\frac{q_{m}C}{K_{d}+C}$$

(2) $$C=q_{m}\frac{C}{q}-K_{d}$$

where *C* (mg/mL) is the equilibrium concentration of β2-m; *q* (mg/mL) is the adsorption capacity when the adsorption reaches equilibrium; *q_m_* (mg/mL) represents the maximal adsorption capacity of the gel and *K_d_* represents the Langmuir adsorption constant.

### 3.7. Specific Adsorption

The immunoadsorption procedure was performed on the AKTA Purifier 100 (GE Healthcare; Chiltern, Buckinghamshire, UK). Human serum used in this experiment was supplemented with 1 mg/mL purified β2-m provided by Crown Medical-Tech (Dalian, China) Co., Ltd. The mixture (2 mL) was applied to one milligram of the immunoadsorptive gel, which was filled in a cylindrical shell and previously equilibrated with 10 mL PBS (10 mM, pH 7.4) at a linear flow rate of 150 cm/h. Non-specifically bound components were washed out with PBS (Phosphate buffer) containing 1 M NaCl. Bound β2-m was then eluted with either 1% SDS (sodium dodecyl sulfate) or 20 mM glycine-HCl (pH 2.0). The fraction eluted from the gel was then analyzed by SDS-PAGE.

An in vitro study was performed to evaluate the adsorption performance of the VHH-based immunoadsorption to major proteins in blood. Five volumes of the serum were incubated with 1 volume of the adsorbent for 2 h at room temperature. The native CL-6B matrix without VHH-immobilization served as a blank control group. The concentration of total protein was determined by biuret method. The specific combination of the bromocresol green (BCG) with serum albumin made use of assaying for serum albumin. The concentrations of other components in serum before and after the adsorption equilibrium were determined by immunoturbidimetry or immunohistochemical analysis.

### 3.8. Storage Stability of the VHH-Based Immunoadsorbent

The immunoadsorbent was preserved in 10 mM PBS supplemented with 0.01% sodium azide at 4°C for different periods of time (0, 15, 30 day). The concentration of VHH released from the gel was measured by a BCA method. The β2-m adsorption capacity was determined by adsorption isotherm.

## 4. Conclusions

To our knowledge, this was the first time that a VHH-based adsorbent was fabricated by one-step method for the extracorporeal removal of β2-m from serum. Under the optimized conditions, we confirmed that the VHH antibody prepared immunoadsorbent was a promising candidate to overcome the problems concerning the specificity and affinity of β2-m. Meanwhile, the bacterial fermentation versus mammalian cell culture (monoclonal antibody-based immunoadsorbents) made the fabrication of the VHH-based immunoadsorbent more cost-effective. Anti-β2-m VHHs expressed in *Escherichia coli* had the required affinity to lower β2-m concentrations to normal levels. All these results showed the feasibility of the VHH-based immunoadsorbent to get rid of β2-m from plasma. Future studies will focus on the search for versatile immobilization chemistries to achieve a higher VHH-coupling and β2-m binding site density.

## Abbreviations

VHH	variable region of the heavy chain antibody
FGE	formylglycine-generating enzyme
β2-m	β2-microglobulin
scFv	single-chain variable fragment
HSA	human serum albumin
